# Comparison of respiratory morbidity between present and ex-workers of quartz crushing units: Healthy workers’ effect

**DOI:** 10.4103/0019-5278.75695

**Published:** 2010

**Authors:** Rajnarayan R. Tiwari, Raj Narain, Y. K. Sharma, Sunil Kumar

**Affiliations:** National Institute of Occupational Health, Ahmedabad, India

**Keywords:** Silicosis, quartz stone workers

## Abstract

**Background:**

Quartz stone grinders are one such group of workers who are exposed to silica and thereby at risk of developing silicosis. However due to increased campaigning against silicosis the scenario has changed.

**Objectives:**

To compare the respiratory morbidities among the present quartz stone workers and the ex-quartz stone workers who have left the job.

**Materials and Methods:**

This cross-sectional study included, 134 ex-workers and 182 current workers of quartz grinding units. All these subjects were subjected to chest radiography and pulmonary function tests.

**Results:**

For 134 ex-workers, the mean age was 31.77 ± 9.99 years and the mean duration of exposure was found to be 2.74 ± 1.65 years while for the present workers, the mean age was 26.74± 7.12 years while the mean duration of exposure was 1.36 ± 2.68 years. The study revealed silicosis in 24 (17.9%), radiological suspected tuberculosis in 17 (12.7%) and silico-tuberculosis in 33 (24.7%) ex-workers while in present workers, radiological suspected tuberculosis in 10 (5.5%) subjects and silicosis grade 1/1 in one subject were found. Among the ex-workers, 14 (10.4%) had a combined type of pulmonary function impairment while 8 (6.0%) and 28 (20.9%) were having restrictive and obstructive type of pulmonary impairments, respectively. Among the present workers, pulmonary function testing revealed the combined type of functional impairment in 1 (0.5%), restrictive type in 13 (7.1%), and obstructive type of functional impairment in 17 (9.2%) subjects.

**Conclusion:**

The high prevalence of respiratory morbidity in ex-workers as compared to current workers can be attributed to the out-of-the-job healthy workers’ effect.

## INTRODUCTION

Pneumoconiosis constitutes the major proportion of the occupational diseases and is one of the ancient occupational diseases. Since Ramazzini first described this group of respiratory disorders among the coal workers,[1] numerous studies have been carried out among workers of various occupations exposed to the various types of dust by virtue of their occupation. However, the silica dust, which is ubiquitous in the atmosphere, still outnumbers the other types of dust, thus making silicosis the most frequently occurring pneumoconiosis.[1–4] The occupations, which expose workers to silica dust, include sandstone quarry, agate industry, slate pencil cutting industry, ceramic and pottery industry, and many more.

Quartz stone grinders are one such group of workers who are exposed to silica. These quartz stone grinders work as an unorganized sector and thus do not come under the purview of social security schemes meant for health and welfare of workers. These workers are engaged in making quartz powder from quartz stone, and it contains 100% free silica.[5] This powder is used as a raw material for glass making, in ceramics, and in the manufacture of chemical filters. The quartz stone is first put into a jaw crusher where a large stone is broken into smaller pieces, which are then taken through the conveyor belt to the disintegrator, which makes the powder out of these small pieces. It is then separated according to its fineness through a vibrating screen and then transported to various industries. All these processes generate a large amount of free silica dust and thus increase the risk of silicosis and silicotuberculosis. In the Godhara region of Gujarat itself, about 10,000 workers work as contract laborers in quartz crushing units.

However, due to increased campaigning against silicosis and efforts to eliminate silicosis, the ground realities have now changed. Fortified by this fact, the present study was carried out to compare the respiratory morbidities among the present quartz stone workers and the ex-quartz stone workers who have left the job.

## MATERIALS AND METHODS

The present study was designed as a cross-sectional study. A total of 134 quartz ex-workers and 182 present or current workers were included in the present study. The ex-workers worked in the same factories in which the present workers are working. These ex-workers were not working since the last 2 years and were residing in the neighboring village. The voluntary organization randomly selected these ex-workers. The present or the current workers were brought by the factory owners for the study. During the period of study, 18 stone quartz units were operational and 185 workers were employed in these units. All the workers were included for the study. However, due to non-cooperation from three workers, the final sample of present workers was 182. Using interview technique as a tool for data collection, demographic and occupational details of the subjects were recorded on the predesigned proforma. Standard diagnostic criteria (ILO Classification of Radiographs for Pneumoconiosis)[6] were used for diagnosing silicosis and silicotuberculosis. The pulmonary functions of the subjects were measured using Spirovit SP-10 (Maker Schiller AG, Switzerland). After calibrating the spirometer according to the procedure given in the catalog, three readings of each ventilatory function of each subject were taken. The readings showing the highest value were recorded considering that the subject had cooperated at his/her best and used for further analysis. Statistical analysis was carried out using the statistical software package “Epi Info 5” (World Health Organization, Geneva, Switzerland) and included the calculation of proportion and percentages and application of tests of significance such as t-test and Chi-square test.

## RESULTS

Table 1 shows the distribution of study subjects according to the demographic characteristics. Majority of the subjects belonged to the age group of 20–39 years. For 134 ex-workers, the mean age was 31.77 ± 9.99 years while for the present workers, the mean age was 26.74 ± 7.12 years. In the present workers’ group, there were fewer females (13.7%) as compared to the ex-workers’ group (44.8%). A total of 53.4% of present workers and 85.1% of ex-workers were illiterate while only 14 present workers were having educational qualification of graduation and above. Mostly these included the supervisory staff.

**Table 1:** Distribution of study subjects according to demographic characteristics

Characteristics	Present workers	Ex-workers
Age (years)		
<20	12 (6.6)	14 (10.4)
20–29	109 (59.9)	40 (29.8)
30–39	47 (25.8)	49 (36.6)
≥40	14 (7.7)	31 (23.2)
Sex		
Male	157 (86.3)	74 (55.2)
Female	25 (13.7)	60 (44.8)
Educational status		
Illiterate	97 (53.4)	114 (85.1)
Primary	15 (8.2)	9 (6.7)
Middle school	43 (23.6)	10 (7.3)
Secondary and higher secondary	13 (7.1)	1 (0.7)
Graduate and above	14 (7.7)	–

All these workers belonged to lower socioeconomic strata according to modified Kuppuswamy’s socioeconomic classification.[7]

Table 2 depicts the distribution of study subjects according to the occupational characteristics. Most of the present workers were working for a period of less than 1 year while majority of the ex-workers worked for 1–5 years. The mean duration of exposure was found to be 2.74 ± 1.65 years for the ex-workers while for the present workers, it was 1.36 ± 2.68 years. More than half of the present as well as ex-workers were working in the crushing process.

**Table 2:** Distribution of study subjects according to occupational characteristics

Characteristics	Present workers	Ex-workers
Duration of exposure (years)		
<1	127 (69.8)	1 (0.7)
1–5	34 (18.6)	126 (94.0)
≥5	21 (11.6)	7 (5.3)
Process		
Crushing	93 (51.1)	92 (68.6)
Screening	15 (8.2)	7 (5.3)
Loading/unloading	24 (13.2)	30 (22.4)
Maintenance	20 (10.9)	3 (2.2)
Others	30 (16.5)	2 (1.5)

The distribution of study subjects according to the respiratory symptoms is shown in Table 3. Cough and dyspnea were the most common symptoms for both the group of workers. However, more of the ex-workers were symptomatic as compared to the present workers.

**Table 3:** Distribution of study subjects according to respiratory symptoms

Respiratory symptoms	Present workers	Ex-workers
Cough	46 (25.0)	38 (20.8)
Phlegm production	24 (13.0)	31 (17.0)
Hemoptysis	3 (1.6)	32 (26.0)
Dyspnea	45 (24.5)	128 (93.4)
Chest pain	36 (19.6)	127 (92.7)
Loss of weight	7 (3.8)	69 (50.4)
Evening rise of temperature	2 (1.1)	48 (35.3)
Loss of appetite	5 (2.7)	52 (38.0)

Table 4 shows the respiratory morbid conditions as diagnosed using ILO Classification of Pneumoconiosis while the distribution of study subjects according to the pulmonary function status after adjusting for respiratory morbidities is depicted in Table 5.

**Table 4:** Distribution of study subjects according to respiratory morbid conditions

Respiratory morbid condition	Present workers	Ex-workers
Silicosis	1 (0.5)	24 (17.9)
Silicotuberculosis	–	33 (24.6)
Tuberculosis	10 (5.5)	17 (12.7)
Normal	171 (94.0)	60 (44.8)

**Table 5:** Distribution of study subjects according to the respiratory function after adjusting for respiratory morbidities

Respiratory function	Present workers	Ex-workers	P-value
Mean spirometric values			
FEV_1_	2.95 ± 0.72	2.79 ± 0.69	>0.05
FVC^1^	3.49 ± 0.76	3.32 ± 0.82	>0.05
PEFR	6.25 ± 1.88	5.74 ± 1.72	>0.05
MMEFR_25-75_	2.89 ± 1.25	3.23 ± 1.18	>0.05
Pulmonary function status			
Normal	142 (82.5)	47 (78.3)	>0.05*
Combined	4 (2.3)	2 (3.3)	
Restrictive	12 (16.7)	3 (5.0)	
Obstructive	14 (8.1)	8 (13.3)	

* Normal PFT status vs. abnormal PFT status; χ^2^ = 0.46, df = 1, nonsignificant.

## DISCUSSION

The present study was carried out among the ex-workers and the present workers of quartz stone crushing units of Godhara, Gujarat.

The mean age of both the groups of workers suggests that the workers were young, and if young workers are exposed to silica dust, they develop respiratory conditions like silicosis and they have to spend their whole life with a disability.

Most of the ex-workers worked for a period of 1–5 years while the present workers worked for a considerably less duration. The mean duration of exposure for the present workers was also significantly less than that of ex-workers. This could be attributed to three reasons. First, due to active campaigning against silicosis through awareness programs and health examination surveys by the National Institute of Occupational Health, now the factory owners have become aware of the fatal disease and its occurrence through exposure to silica dust. Thus, they are now frequently changing the workers. Second, this finding could be due to healthy workers’ effect, which can again be due to two reasons: either the sick subjects have left the job or because of the awareness, the employers might have sent the healthy workers for the study. Third, this could be due to the modification in the machines for less dust generation as suggested by us. This was authenticated by the effective dust reduction up to 60% at a source on introduction of the new control technology.

The cough, dyspnea, and chest pain were the common symptoms as reported by the study subjects for both the groups. The difference was statistically significant (χ^2^ = 121.4, df = 1, *P*<0.001). However, more of ex-workers were symptomatic as compared to present workers. This could be due to the higher duration of exposure to the silica dust among ex-workers as compared to present workers. When adjusted for age, the complaints were more in those aged ≥ 30 years for present workers as well as ex-workers. Similarly, after adjustment for sex, more males were found symptomatic as compared to females in both groups. When analyzed after adjusting for the duration of exposure, those exposed for ≥1 year were more symptomatic as compared to those who worked for lesser duration.

Using ILO Classification for Pneumoconiosis for the diagnosis of silicosis and silicotuberculosis, only 1 case of silicosis of profusion grade 1/1 and 10 cases of tuberculosis were found in the present workers’ group while in the ex-workers’ group, 24 (17.9%) cases of silicosis, 33 (24.6%) cases of silicotuberculosis, and 17 (12.7%) cases of tuberculosis were found. A total of 94% of present workers and 44.8% of ex-workers were free from any respiratory morbidity. This difference was found to be statistically significant. This could be attributed to the high duration of exposure among ex-workers. This could also be attributed to the healthy workers’ effect among the present workers. After adjusting for respiratory morbidities and using the Harwarth’s criteria[7] for the categorization of lung function impairments into restrictive, obstructive, and combined type, 82.5% of the present workers were having a normal pulmonary function while only 78.3% of the ex-workers were normal. This suggests a greater prevalence of respiratory abnormalities in the ex-workers as compared to present workers. Though this difference was statistically nonsignificant (χ^2^ = 0.46, df = 1, *P*>0.05), this could be related to the dose of the dust inhaled as measured indirectly by the duration of exposure, which was high in ex-workers. The mean levels of FVC, FEV_1_, PEFR, and MMEFR_25-75_ were also statistically nonsignificantly lower in the ex-workers’ group as compared to present workers. In both the groups, the measurements of these spirometric parameters were lower among those having respiratory morbidity as compared to those free from that. Earlier studies have also reported similar findings.[8–12]

The present study suggests that the respiratory morbidities were high in the ex-workers. This can be attributed to the healthy workers’ effect. The job of quartz crushing is a highly laborious job and requires a high degree of physical fitness. However, the presence of respiratory morbidity compels these workers to leave the job thereby leaving only healthy workers at workplace. Also, due to a low literacy level these workers fail to understand the importance of preventive measures and thereby suffer more. Thus, there is a need to increase the awareness regarding the fatal nature of disease and the effective preventive measures among all stakeholders.
